# Application of Green Polymeric Nanocomposites for Enhanced Oil Recovery by Spontaneous Imbibition from Carbonate Reservoirs

**DOI:** 10.3390/polym15143064

**Published:** 2023-07-17

**Authors:** Yaser Ahmadi, Mohamed Arselene Ayari, Meysam Olfati, Seyyed Hossein Hosseini, Amith Khandakar, Behzad Vaferi, Martin Olazar

**Affiliations:** 1 Chemical and Petroleum Engineering Department, Ilam University, Ilam 69315516, Iran; 2 Department of Civil and Architectural Engineering, Qatar University, Doha 2713, Qatar; 3 Technology Innovation and Engineering Education Unit, Qatar University, Doha 2713, Qatar; 4 Department of Electrical Engineering, Qatar University, Doha 2713, Qatar; 5 Department of Chemical Engineering, Shiraz Branch, Islamic Azad University, Shiraz 7198774731, Iran; 6 Department of Advanced Calculations, Chemical, Petroleum, and Polymer Engineering Research Center, Shiraz Branch, Islamic Azad University, Shiraz 7198774731, Iran; 7 Department of Chemical Engineering, University of the Basque Country (UPV/EHU), P.O. Box 644-E48080 Bilbao, Spain; martin.olazar@ehu.eus

**Keywords:** green nanocomposites, walnut, eucalyptus, salinity, wettability, polymer

## Abstract

This study experimentally investigates the effect of green polymeric nanoparticles on the interfacial tension (IFT) and wettability of carbonate reservoirs to effectively change the enhanced oil recovery (EOR) parameters. This experimental study compares the performance of xanthan/magnetite/SiO_2_ nanocomposites (NC) and several green materials, i.e., eucalyptus plant nanocomposites (ENC) and walnut shell ones (WNC) on the oil recovery with performing series of spontaneous imbibition tests. Scanning electron microscopy (SEM), X-ray diffraction (XRD), energy-dispersive X-ray spectroscopy (EDAX), and BET (Brunauer, Emmett, and Teller) surface analysis tests are also applied to monitor the morphology and crystalline structure of NC, ENC, and WNC. Then, the IFT and contact angle (CA) were measured in the presence of these materials under various reservoir conditions and solvent salinities. It was found that both ENC and WNC nanocomposites decreased CA and IFT, but ENC performed better than WNC under different salinities, namely, seawater (SW), double diluted salted (2 SW), ten times diluted seawater (10 SW), formation water (FW), and distilled water (DIW), which were applied at 70 °C, 2000 psi, and 0.05 wt.% nanocomposites concentration. Based on better results, ENC nanofluid at salinity concentrations of 10 SW and 2 SW ENC were selected for the EOR of carbonate rocks under reservoir conditions. The contact angles of ENC nanocomposites at the salinities of 2 SW and 10 SW were 49 and 43.4°, respectively. Zeta potential values were −44.39 and −46.58 for 2 SW and 10 SW ENC nanofluids, which is evidence of the high stability of ENC nanocomposites. The imbibition results at 70 °C and 2000 psi with 0.05 wt.% ENC at 10 SW and 2 SW led to incremental oil recoveries of 64.13% and 60.12%, respectively, compared to NC, which was 46.16%.

## 1. Introduction

Due to the low level of oil production by standard methods, studies have been approached based on the use of alkaline [1,2], polymer [3,4], and surfactant [5,6] chemicals to improve oil recovery. Changing brine composition in the injected water is known as a cost-effective and efficient method, which can affect EOR scenarios [7]. One of the standard plans to intensify the oil recovery in all types of reservoirs is the injection of low concentrations of brine, known as low salinity flooding [8]. The main advantages of flooding with low salinity are its lower cost compared to expensive chemical methods and the fact that no extra materials are required for flooding with low salinity [9]. Changing water composition in intense salinity flooding is a fascinating topic, and researchers have focused on it to maximize oil recovery [10,11]. Therefore, EOR methods with different water flooding methods have been recently used by changing water composition, that is, changing salinity or adding nanoparticles to the water to decrease residual oil saturation [8]. The small size and high specific surface area of nanoparticles are favorable characteristics to increase EOR through changes in the involved parameters [12,13,14], such as IFT [15] and CA. Studies dealing with changes in these parameters in the presence of novel nanoparticles are highly relevant. Furthermore, given their low concentration required, they are known as cost-effective materials with many applications in petroleum engineering [16,17].

Nanoparticles such as alumina [18,19], silica [20,21], nickel oxide [22], silver [23], and magnetite [24,25] have recently been used in different processes, including enhanced oil recovery. For example, silica nanoparticles have been used in the sandstone reservoir as they effectively change the main parameters in porous media, such as IFT and CA [26]. They used Cedr extraction and silica nanoparticles by obtaining 5 wt.% as the CMC point for surfactant, and it was found that, as silica nanoparticles concentrations were increased from 0 to 2500 ppm, IFT was decreased from a base point of 15.5 to 13 mN/m. Moreover, it was seen that, due to increasing surfactant concentration from 0 to 10 wt.%, IFT decreased 35 to 11.9 mN/m. Maleki et al. (2023) used a combination of silica/alumina nanocomposites based on polyacrylamide for EOR to obtain reservoir parameters, such as IFT. It was found that IFT reduction was the most important parameter which control nanocomposite behavior. Based on the results, at different salinity of 250,000 ppm to 180,000 ppm, oil recovery of 95.83 and 70.33% were obtained [27]. Xanthan is another essential and environmentally friendly biopolymer with many applications in the oil industry. Fu et al. (2022) showed that xanthan efficiently increases ultimate recovery due to salt resistance properties, rheological properties, great thickening, and improvement in viscosity [28]. Modified Xanthan in their study increased EOR 7% more than base Xanthan due to increasing viscosity up to three times. Nanoparticles produce positive changes in the porous environment, and they can easily travel through low-porosity media; salinity is an essential factor that must be studied in detail [29]. Navaie et al. (2022) used Xanthan gum as a natural surfactant for EOR [30]. It was concluded that the contact angle was decreased from 60.52 to 16.71° in the carbonate reservoir and 50.1 to 8.87° in the sandstone reservoirs. Moreover, it was observed that oil recovery was increased by up to 28.6%, and IFT was decreased by up to 74% in the presence of solution. Ali et al. (2019) used a combination of silica and xanthan (ZnO/SiO_2_/xanthan) nanocomposites to modify the main parameters involving EOR (IFT and CA), and based on their results, the above two parameters are significantly improved in the presence of nanocomposites [20]. Based on their results, at 2000 ppm, IFT decreased from 31.8 to 2.016 mN/m, and the recovery factor increased from 46.96% to 66.24%. Motraghi et al. (2023) used silica/KCl/Xanthan to check IFT behavior [31]. Different solvent concentrations of 5%, 10%, 15% and at 25, 50, and 75 °C, and it was found that 1000 ppm of nanocomposites at 75 °C were the optimum value for IFT reduction. The presence of ferrofluids, such as magnetite nanoparticles, could also influence the parameters involving EOR; that is, they reduce viscosity and shear stress. Aristizábal-Fontal et al. (2018) showed that viscosity and shear stress were decreased by up to 81% and 78%, respectively, in heavy oil [32]. Magnetite nanoparticles have wide applications in the oil industry, especially in problematic wells and conditions involving asphaltene precipitation. Betancur et al. (2016) reported that magnetite can adsorb asphaltene up to 61 mg/g, and decrease asphaltene aggregation by up to 39% [33]. Recently, the use of green nanomaterials has found high popularity as they are safe from the environment and human health perspectives [34,35]. Furthermore, principles of green chemistry help synthesize nanomaterials without toxic components [36] and save energy and renewable inputs [37]. Green technology also means using nanomaterials to make the current production procedures and their products in an environmentally friendly way. Recently, our team developed a new polymeric material of green nanocomposites (Xanthan/SiO_2_/ZnO) for EOR based on the main parameters of this process (IFT and CA) at different salinity conditions. It was observed that green nanocomposites have high efficiency for EOR from carbonate reservoirs at different salinity conditions. Thus, CA and IFT were reduced to significantly lower values than those corresponding to commercial silica, and oil recovery at 2 SW and 10 SW salinity conditions increased to 25.1% and 34.1%, respectively [38]. Ali et al. (2020) utilized magnetite/xanthan/silica for the wettability alteration and IFT reduction, with xanthan being synthesized from the Alocasia macrorrhiza plant. The application of 250 to 1500 ppm concentration of the materials results in wettability alteration from the strongly oil-wet to the water-wet in the carbonate reservoir, and IFT decreased in the presence of nanocomposites [24]. According to their results, IFT was decreased from 28.3 to 4.35 mN/m, and contact angle was decreased from 134 to 28° after using nanocomposites. As it was mentioned, the main effective parameters are IFT and CA reduction, and Table 1 summarized the last works for EOR in the presence of nanoparticles.

It should be noted that although many EOR studies have already been conducted, there are limited works covering recovery tests at reservoir conditions. Moreover, too high concentrations have been commonly used for low permeability carbonate, and the mentioned plant is not easily accessible, especially in our region. Therefore, more accessible and cost-effective materials (i.e., eucalyptus plant and the walnut shell) are selected in this study instead of the Alocasia macrorrhiza plant, and their performance is compared with the base material (silica/xanthan/magnetite). Accordingly, the main novelty of this work was using cost-effective polymeric nanocomposites at different salinity at reservoir conditions. 

## 2. Materials and Method

### 2.1. Materials

**Figure 1:**
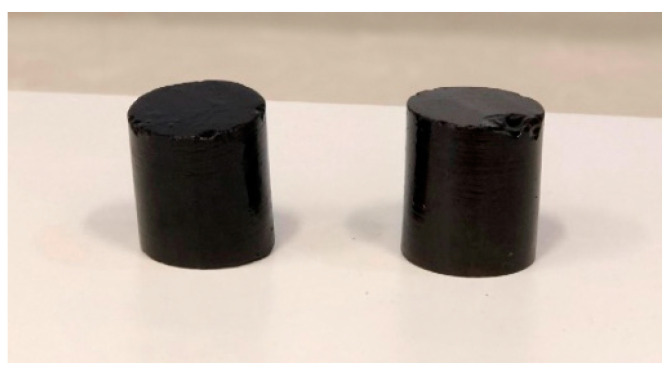
Plugs for performing imbibition tests.

The core sample and crude oil are provided by one of Iran’s carbonate oil reservoirs. Core porosity, permeability, and oil density and viscosity were 14.90%, 10.45 mD, and 10.00 cP, respectively. It was tried to select two same plugs for performing imbibition tests in the presence of nanocomposites, as shown in Figure 1. Initial carbonate plug mass (gram), plug length (cm), diameter (cm), and saturated oil plus mass (gram) were 112.20, 4.80, 3.80, and 119.20, respectively. 

Solvents, salts (NaCl, CaCl_2_, MgCl_2_, KCl), and other chemical compounds were supplied by Merck and Aldrich with a purity of 99.5%. Xanthan gum (98% purity) was supplied by Aldrich company, iron chloride (FeCl_3_), and sodium metasilicate (Na_2_SiO_3_) with 98% purity by Merck company. The values of brine composition, pH, and density are shown in Table 2 and Table 3, respectively, according to different salinities. Distilled water was used during dilution. After the preparation of specific amounts of salts (FW, SW, 2 SW, and 10 SW), the solution was prepared with DIW, and it was homogenized before applying.

### 2.2. Synthesizing Green Polymeric Nanocomposites (NC, WNC, and ENC) 

**Figure 2:**
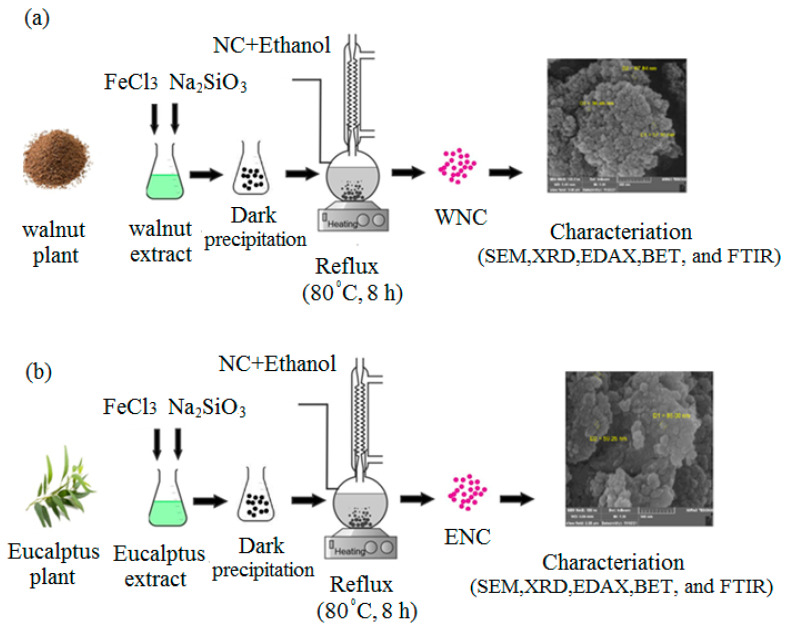
Details about the preparation of (**a**) Walnut and (**b**) Eucalyptus nanocomposites.

The based material was xanthan/magnetite/SiO_2_, and it was obtained based on Ali. et al., 2020 [24]. At the same condition, the desired plant extracts (from walnut bark or eucalyptus leaves) were prepared by heating 50 g of the sample (walnut bark powder or dried eucalyptus leaves) in 400 cm^3^ of distilled water at 80 °C for 40 min. Then, the samples obtained from both extracts were filtered, 2 g of FeCl_3_ and 5 g of Na_2_SiO_3_ were added to 100 mL of the liquid solution at 80 °C, and the mixture was stirred at a pH value of 10 by adding NaOH. The stirring stops just after the formation of a black precipitate, and the solution filters for separation. The possible impurities are removed by heating the obtained residue at 400 °C and washing it with hot distilled water. Finally, the nanocomposites are synthesized by mixing 15 g of xanthan gum, 200 cm^3^ of ethanol, and the collected sediment under reflux conditions for 8 h at 80 °C. Figure 2a and Figure 1b show details about the preparation of WNC and ENC nanocomposites.

### 2.3. CA, IFT, and Imbibition Tests

**Figure 3:**
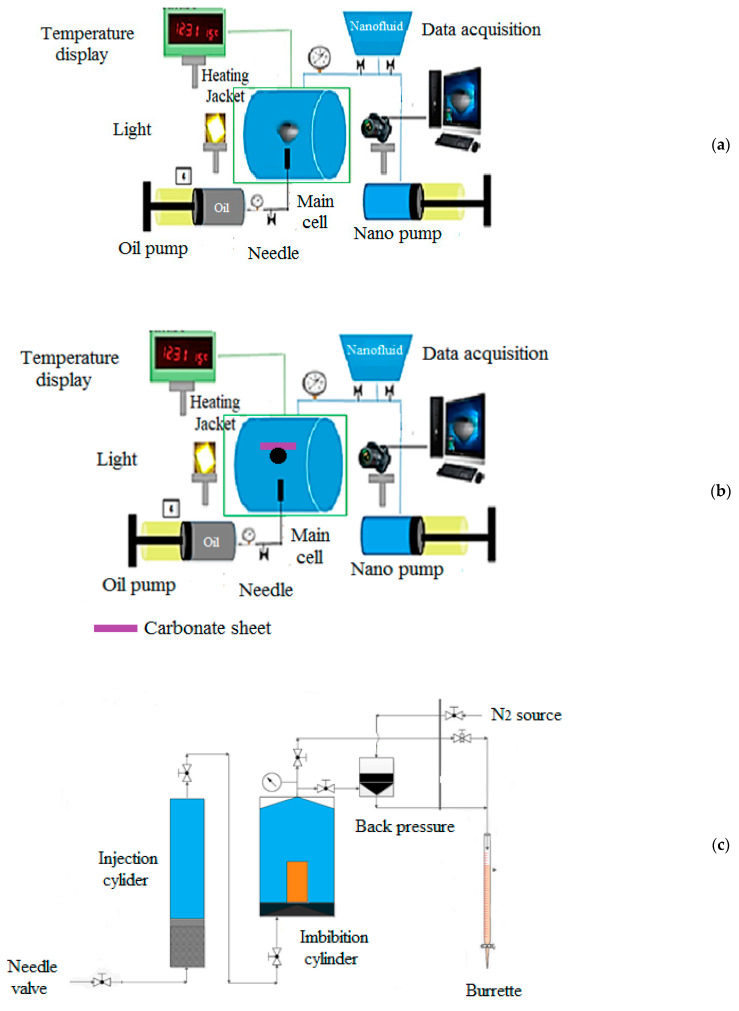
Equipment for (**a**) IFT, (**b**) CA, and (**c**) Imbibition tests.

A schematic view of the equipment for IFT and CA tests is shown in Figure 3a and Figure 2b, respectively, with the Petro AZMA model. After preparing the nanofluids at different salinities, they were transferred to the primary cell. Next, the crude oil was injected with an oil pump, and the drops were recorded with a camera. The system pressure was set to 2000 psi with a nano pump, and the temperature was 70 ◦C in all tests. Finally, drops were calculated based on their analysis and IFT software. Instructions were rigorously followed for CA tests, and they differed only in the droplets on the carbonate sheets [38]. Figure 3c shows a schematic view of the equipment for the imbibition test, which was used for ascertaining oil recovery. Nanofluids containing 0.05 wt.% ENCs were prepared at two different salinities of 2 SW and 10 SW for IFT and CA tests, and the fluid was transferred to the primary cell surrounding the saturated oil core. The core initial mass, oil sodden group, initial oil saturation, and oil volume were recorded in the imbibition tests. Cell pressure and temperature were set at 70 °C and 2000 psi, and runs were performed with 2 SW and 10 SW salinities. All experiments in our study were repeated three times, and their average values were reported.

## 3. Results and Discussion

### 3.1. Chemical Composition and Surface Morphology of Nanocomposites

**Figure 4:**
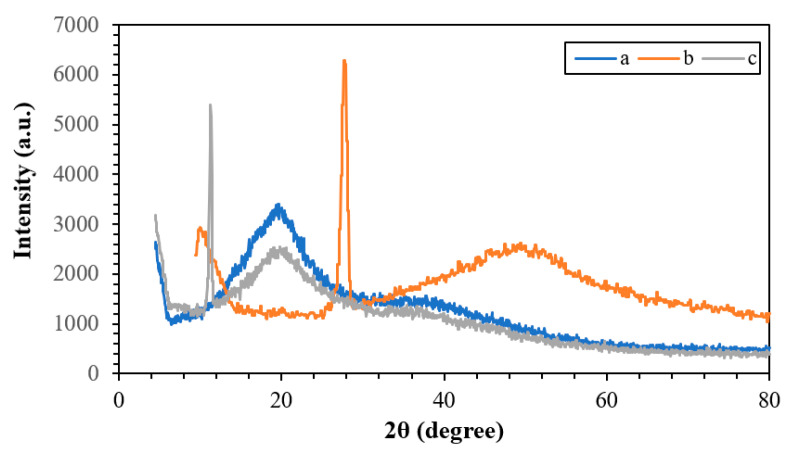
XRD spectrum of the (**a**) NC, (**b**) ENC, and (**c**) WNC.

X-ray diffraction analysis was applied to observe the crystal structure with the PW1730 model. Figure 4 presents the X-ray diffraction spectrum of the base NC and those corresponding to WNCs and ENCs. As observed, the XRD pattern of the sample without the extract is different from that containing the extract, which is evidence of the successful preparation of these compounds. The 2ϴ peaks at 11.32 and 27.82 correspond to eucalyptus plant and walnut shells, respectively [44,45,46]. 

**Figure 5:**
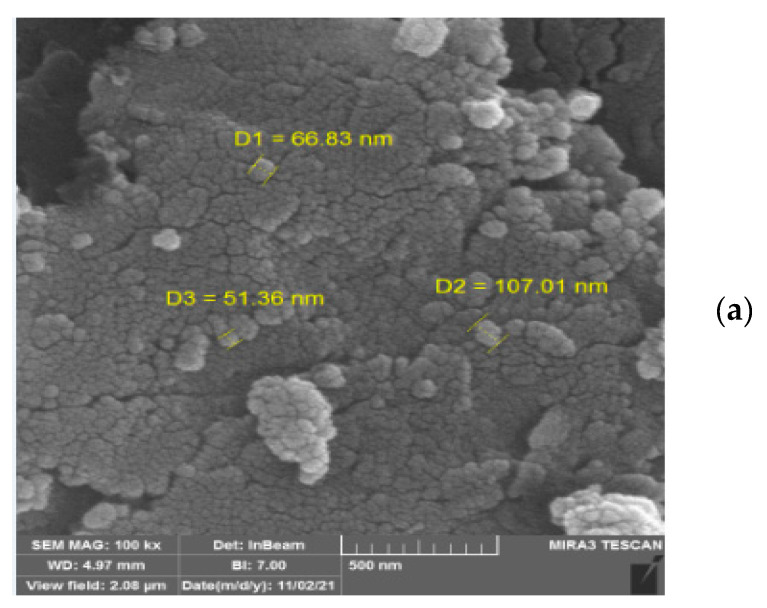

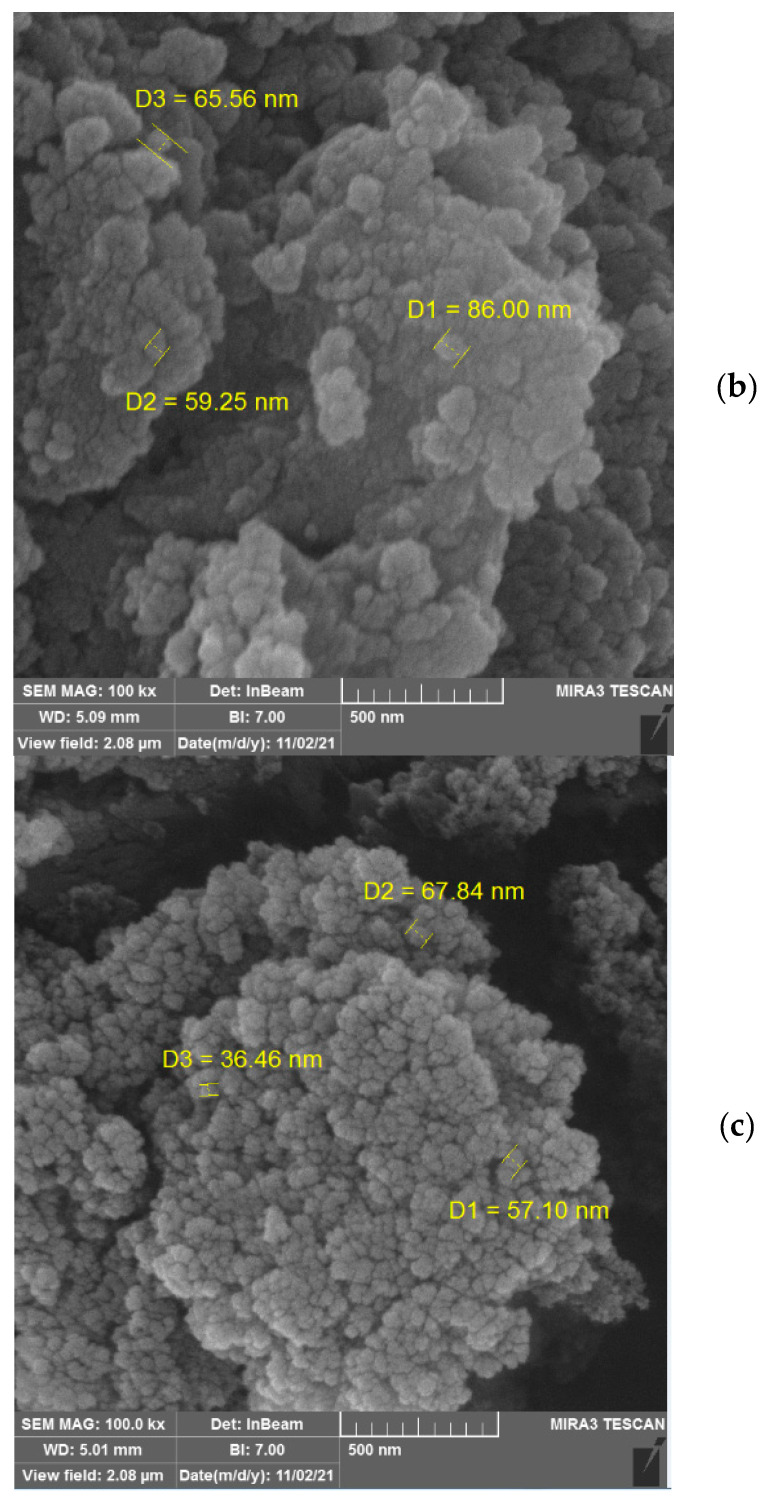
SEM images of (**a**) NC, (**b**) ENC, and (**c**) WNC.

Figure 5a–c introduce the scanning electron microscope images of NCs, ENCs, and WNCs, respectively. The morphological images show that the base NCs are spherical with a dimension range from 51 to 107 nm, and those modified with eucalyptus extract (ENCs) and walnut extract (WNCs) have average diameters of 59–86 nm and 36–67 nm, respectively.

**Figure 6:**
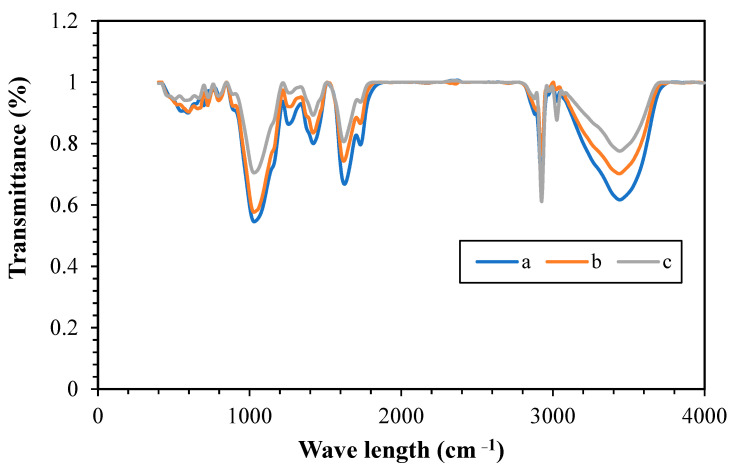
FT-IR spectrum of (**a**) NC, (**b**) ENC, and (**c**) WNC.

Figure 6 depicts the FT-IR spectrum of NC, ENC, and WNC nanocomposites which were performed with the FTIR setup model Thermo AVATAR. Based on the results, the peaks that appeared at 3400 cm^−1^ are associated with O–H and N–H stretching vibrations, and those at 2925, 1750–1650, and 1420 cm^−1^ are related to C–H stretching vibrations, C=O carbonyl groups, and C=C carbonyl groups, respectively. Furthermore, stretching vibrations related to C–O appeared at 1030 cm^−1^.

**Figure 7:**
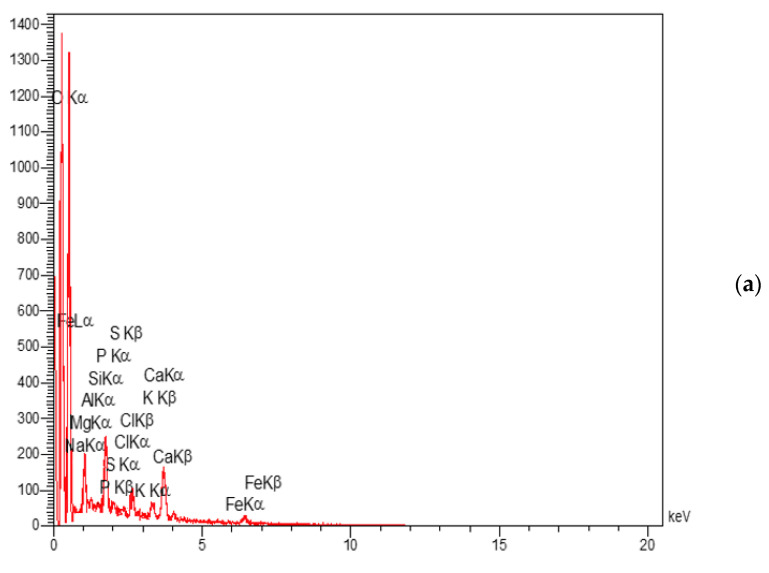

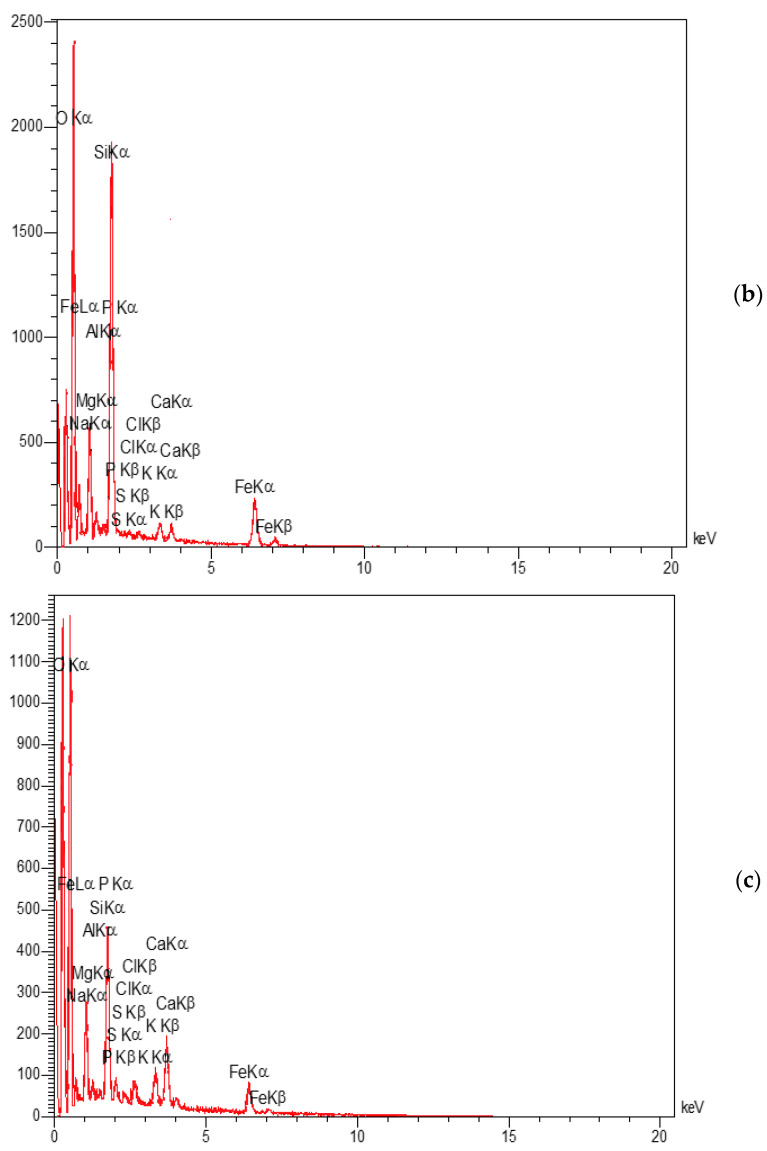
EDAX spectra of (**a**) NC, (**b**) ENC, and (**c**) WNC.

Energy Diffraction X-ray analyses (EDAX) were performed with the TESCAN MIRA2 model to investigate the elemental analysis of nanocomposites. EDAX tests for NC, ENC, and WNC nanocomposites are illustrated in Figure 7a, 7b, and 7c, respectively. Their elemental analysis is set out in Table 4. By examining the X-ray energy diffraction spectra, the presence of new elements corresponding to the plant extracts confirms that these nanoparticles have been correctly synthesized.

**Figure 8:**
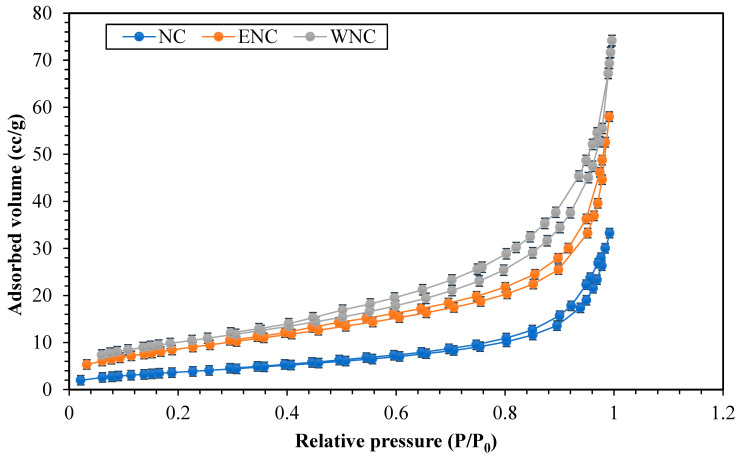
N_2_ adsorption–desorption isotherms of NC, ENC, and WNC samples.

Figure 8 shows the isotherms of the N_2_ adsorption–desorption of NC, ENC, and WNC samples determined based on the BET (Brunauer–Emmett–Teller) method with the BELSORP MINI II model. Table 5 reports the pore volume, average pore diameter, and BET surface area. The specific surface areas of NC, ENC, and WNC samples were 14.22, 36.04, and 30.97 m^2^/g, respectively. Adding eucalyptus (ENCs) and walnut shells (WNCs) increased the pore volume of the nanocomposites, as well as their specific surface area. 

### 3.2. Results of the IFT, CA, and Imbibition Tests

**Figure 9:**
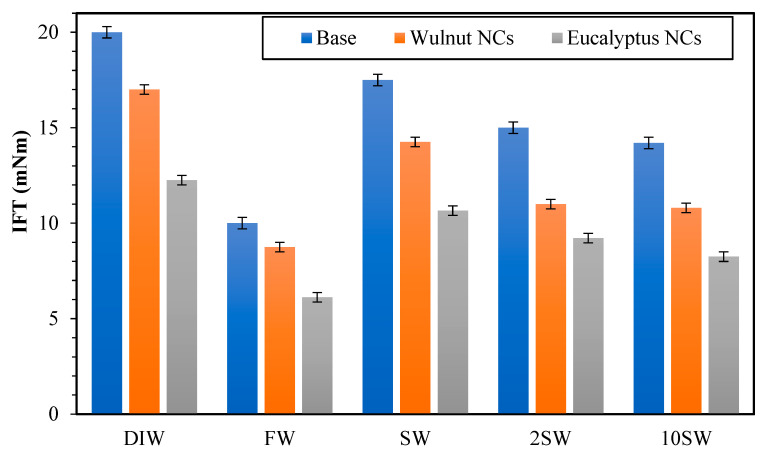
IFT of NC, WNC, and ENC.

One of the crucial parameters to monitor when flooding with low salinity is the interfacial tension. Figure 9 shows the IFT between crude oil and water containing different nanocomposites (NC, ENC, and WNC) at 0.05 wt.% concentration under reservoir conditions (70 °C and 2000 psi). The results show that the interfacial tension decreases when salt water concentration is decreased among SW, 2 SW, and 10 SW, FW had the lowest IFT, and DIW had the highest IFT.

Furthermore, interfacial tension with formation water is surprisingly lower than with the other salty solutions tested. Thus, when the salt water concentration was decreased, the surface-active material particles moved toward the contact surface, and the IFT decreased. As the concentration of saltwater was increased, the surface energy of the particles attenuated in the oil. 

Among SW, 2 SW, and 10 SW, it was found that a solution of lower concentration reduces the solution ionic strength and, therefore, the interfacial tension, which enhances oil extraction [47]. This observation is quite consistent with the results reported by Nowrouzi et al. [48] and Ali et al. [49]. They described the effect of concentration and ionic compound of the seawater and reported that diluting the seawater can effectively reduce the IFT.

As observed in this figure, the IFT values of Magnetite/SiO_2_/Xanthan nanocomposites decrease as the salinity of the solution is decreased. The main fact for IFT reduction lies in the use of xanthan gum in the structure of nanocomposites and the subsequent increase in tensile strength [38,50]. It should be noted that the data of IFT shown in Figure 9 correspond to the average of three replicates.

**Figure 10:**
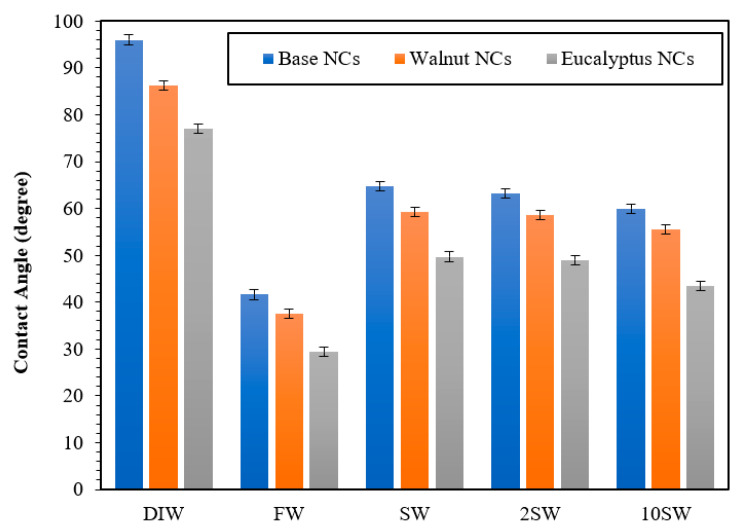
Contact angles for base NC, WNCs, and ENCs at different salinities.

In addition, wettability is another crucial factor of a porous medium. This factor plays a vital role in water displacement and EOR parameters. The rock wettability is possible to change from oil-wet to water-wet in the presence of nanoparticles. An adsorbed layer of nanomaterials on the rock surface affects the fluid/rock as well as fluid/fluid interactions. A wide range of factors, including rock surface characteristics, nanoparticles size/concentration, and the nanoparticles-rocks interaction, determine the type of deposited layer on the rock surface as well as the resulting wettability alteration. Figure 10 shows the contact angles measured in the presence of different nanocomposites (NCs, WNCs, and ENCs) and different salinity levels (DIW, FW, SW, 2 SW, and 10 SW). The results of the runs under the reservoir temperature and pressure (70 °C and 2000 psi) with 0.05 wt.% nanocomposites concentration showed that the injection of ENC and WNC nanocomposites solutions increases the hydrophilic strength of the carbonate rock more than the injection of the base NC nanocomposites. Deionized water has the highest CA changes among other tested points. FW, 2 SW, and 10 SW dilute seawater solutions recorded the lowest wettability in the presence of NC, WNC, and ENC nanocomposites at the concentration of 0.05 wt.% under the temperature and pressure of the reservoir (70 °C and 2000 psi). Furthermore, all the nanocomposite solutions enhance the hydrophilic properties of carbonate rock, but (independently of the salinity) ENC and WNC nanocomposites decreased the CA more than the base NC nanocomposites. Based on these results, the green particle of the highest efficiency was chosen for further studies in the porous media. Thus, the ENC nanocomposites at the salinities of 2 SW and 10 SW were selected for performing imbibition tests. The contact angles of ENC nanocomposites at the salinities of 2 SW and 10 SW were 49 and 43.4°, respectively.

**Figure 11:**
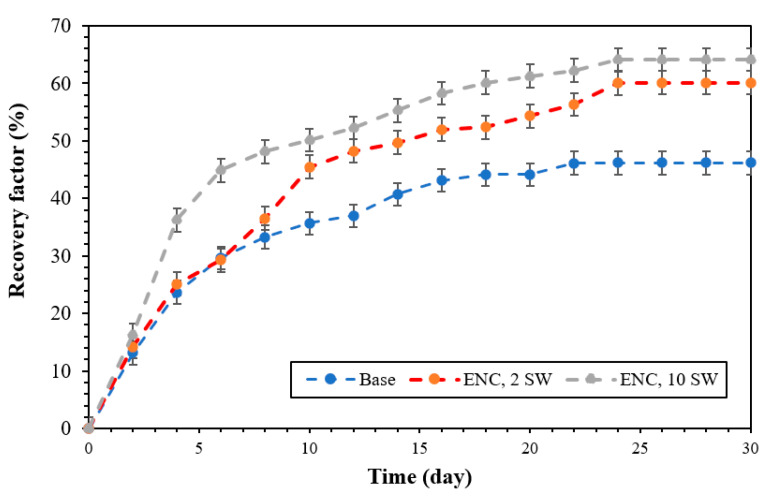
Oil recovery factors for base nanocomposites and ENC.

Figure 11 shows oil recovery test scenarios using base (NC) and ENC at different salinity of 2 SW and 10 SW under 2000 psi and 70 °C by using spontaneous imbibition tests. Oil recovery for the base, 2 SW, and 10 SW were 46.16, 60.12, and 64.13%, respectively. The changes in CA and IFT are responsible for increasing oil recovery with the nanocomposites. Furthermore, a highly relevant fact that must also be considered is stability. Thus, nanoparticle stability is one of the crucial factors that must be considered during flooding, especially in carbonate reservoirs of low permeability. Zeta potential was measured to ascertain stability in the presence of ENC nanocomposites with Malvern Zetasizer Nano ZS ZEN3600, Malvern Instruments Ltd, Malvern, United Kingdom and values of −44.39 and −46.58 were obtained for 2 SW and 10 SW, respectively. This finding clarifies that this nanocomposite is highly stable in the two solutions [51].

Table 6 compares the results of the current study with relevant previous works. Based on the results, nanocomposites had better tertiary oil recovery compared to others.

## 4. Conclusions

In this study, green polymeric nanocomposites of eucalyptus and walnut shell (ENC and WNC) were prepared at different salinity concentrations [DIW, FW, SW, 2 SW, and 10 SW] to enhance oil recovery under reservoir conditions [70 °C and 2000 psi] by spontaneous imbibition tests. The main advantages of this study were using cost-effective green materials at reservoir conditions at a low concentration of 0.05 wt.%. Based on the results of IFT and CA tests, ENC nanocomposites perform better than WNC nanocomposites, and they were selected for actual imbibition tests at 2 SW and 10 SW salinities (they are the most efficient solutions). In the presence of ENC nanocomposites, the zeta potential for 2 SW and 10 SW salinities are −44.39 and −46.58, respectively, which is evidence of the excellent stability of eucalyptus nanocomposites. Furthermore, the wettability of the carbonate rock changes towards hydrophilicity for low concentrations of brine. Two scenarios, i.e., 2 SW and 10 SW, with these eucalyptus nanocomposites led to the best results in wettability tests. The oil recoveries with ENC at 2 SW and 10 SW were 60.12% and 64.13%, respectively, compared to NC, which was 46.16%. The explanation for this result lies in the reduction of IFT and CA and their high stability. Future research should be devoted to ascertaining the influence of the parameters analyzed in this study (solution salinity and reservoir pressure and temperature) when the process is scaled up.

## Figures and Tables

**Table 1:** IFT and CA change in the presence of nanoparticles.

NPs	IFT (mN/N) Clean/with NPs	Contact Angle (°) Clean/with NPs	Ref.
SiO_2_	38.5/1.45	134/82	[39]
Fe_2_O_3_	38.5/2.75	132.5/101	[39]
SiO_2_	38.4/26.5	90/60	[40]
SiO_2_	-	135.5/66	[41]
SiO_2_	20/1.87	-	[42]
ZnO/SiO_2_/Xanthan	-	79/75 (2 SW)	[8]
ZnO/SiO_2_/Xanthan	-	78/74 (10 SW)	[8]
SiO_2_	37.5/22.1	-	[43]
SiO_2_	35/10.9	-	[26]

**Table 2:** Brine composition.

Type	Ten Dilute SW (ppm)	Two Dilute SW (ppm)	SW (ppm)	Formation Water (ppm)
NaCl	2840	14,200	28,400	140,316
CaCl_2_	138	690	1380	40,287
MgCl_2_	643	3215	6430	2856
KCl	80	400	800	800

**Table 3:** pH and density of the brine.

Salinity Type	Density (g/mL)	pH (-)
DIW	1.00	7.00
SW	1.05	6.46
2 SW	1.01	6.93
10 SW	1.00	6.42
FW	1.07	8.11

**Table 4:** EDAX elemental analysis of the nanocomposites.

Elements	NC (%)	ENC (%)	WNC (%)
C	44.78	29.78	43.57
O	47.17	47.63	43.41
Na	1.66	3.92	2.46
Mg	0.30	0.56	0.31
Al	0.17	0.24	0.15
Si	1.37	8.8	2.71
P	0.26	0.19	0.36
S	0.24	0.26	0.27
Cl	0.78	0.28	0.57
K	0.60	0.82	1.31
Ca	1.93	0.65	2.21
Fe	0.74	6.87	2.85

**Table 5:** Textural characteristics of the investigated nanocomposites.

Sample	Surface Area (m^2^/g)	Average Pore Diameter (nm)	Pore Volume (cm^3^ (STP)/g)
NC	14.22	14.29	0.05
ENC	36.04	11.65	0.10
WNC	30.97	11.35	0.08

**Table 6:** Compare tertiary oil recovery with several relevant nanoparticles.

Nanoparticles	Permeability (mD)	Tertiary Oil Recovery (%)	References
ENC at 10 SW	10.45	17.97	Current study
SiO_2_	5.00	16.00	[52]
SiO_2_	0.21	16.00	[53]
SiO_2_/Xanthan	2700	13.18	[54]
KCl/SiO_2_/Xanthan	7.3	17.05	[55]

## Data Availability

The datasets used and/or analyzed in the current study are available from the corresponding author upon reasonable request.
